# Combined use of total glucosides of paeony and hydroxychloroquine in primary Sjögren's syndrome: A systematic review

**DOI:** 10.1002/iid3.1044

**Published:** 2023-10-20

**Authors:** Aiping Zhang, Shilei Chen, Riyang Lin

**Affiliations:** ^1^ Hangzhou Traditional Chinese Medicine Hospital Affiliated to Zhejiang Chinese Medical University Hangzhou China; ^2^ Zhuji Maternal and Child Health Hospital Shaoxing China

**Keywords:** hydroxychloroquine, primary Sjögren*'*s syndrome, total glucosides of paeony

## Abstract

**Objective:**

To assess the effectiveness and safety of the total glucosides of paeony (TGP) combined with hydroxychloroquine (HCQ) on the treatment of primary Sjögren's syndrome (pSS) by conducting a meta‐analysis.

**Methods:**

Eight databases were searched for randomized controlled trials (RCTs) reporting the use of TGP combined with HCQ for pSS, which are before May 10, 2022. Meta‐analyses were performed on disappeared clinical symptoms (dry mouth and dry eyes), Schirmer's test, saliva flow test, erythrocyte sedimentation rate (ESR), index of immunoglobulin G (IgG), immunoglobulin M (IgM), immunoglobulin A (IgA), and adverse events (AEs). The Revman 5.4 software was used for this meta‐analysis.

**Results:**

Seven RCTs which included 632 participants were identified. The pooled results showed significant differences in clinical symptoms disappear (dry mouth and dry eyes) (*p* = .0004), IgM (*p* < .00001), IgA (*p* < .00001), salivary flow rate (*p* < .00001) and Schirmer*'*s test (*p* = .02) in the comparison of TGP combined with HCQ and HCQ alone. For the IgG and ESR, both pooled and subgroup analyses showed that TGP + HCQ was superior to HCQ alone. For the safety analysis, no significant differences in AEs (*p* = .39) was revealed. The more frequently seen adverse reactions were diarrhea, vomit and there was no severe adverse events were reported in TGP + HCQ group.

**Conclusion:**

Therefore, TGP + HCQ can be considered to be a potentially valid and safe combination for the treatment of pSS in the clinic.

## Graphical abstract

We performed a meta‐analysis of the results of seven studies, including 632 patients of primary Sjögren's syndrome. The results showed that the combination of total peony glucoside with hydroxychloroquine (HCQ) had significant advantages over HCQ alone in improving patients' clinical symptoms and reducing immunoglobulin A, immunoglobulin M, and immunoglobulin G levels.

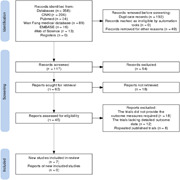

## 1 INTRODUCTION

Primary Sjögren's syndrome (pSS) is a chronic autoimmune disorder primarily and exocrine involvement is the dominant feature, which often leads to such clinical manifestations as xerostomia and keratoconjunctivitis sicca.^1^ At the same time, it can involve multiple systems such as digestion, respiratory, cardiovascular, endocrine, and central nervous system. According to related epidemiological research investigations, the morbidity of pSS in China is the second highest among all rheumatic diseases, which is about 0.77% in the population and pSS is expected to affect more than 10 million Chinese.^2^ Researchers estimate that the prevalence of primary Sjögren syndrome is about 0.5%–4.8%, involving about 1,500,000 to 4,000,000 people in America. Primary Sjögren syndrome is highly insidious and the general population lacks knowledge of this disease, resulting in a higher potential prevalence in the population. For these reasons, currently, the pSS is still considered to be a tricky illness in all chronic autoimmune rheumatic diseases.^3^, ^4^

No treatment has been proved to alter the progression of Sjögren's syndrome, so current treatment options focus on symptom relief (pilocarpine, cevimeline, artificial teardrops, and ointments for helping oral and ocular dryness). Glucocorticoids, immunosuppressants and biologic agents (rituximab) are indicated for the treatment of significant systemic manifestations.^5^ However, most of these medications have no effect on the patient's dry symptoms.^6^ Hydroxychloroquine (HCQ) is an immunomodulatory drug listed as first‐line treatment in The Sjögren's syndrome Foundation Clinical Practice Guidelines, and is usually prescribed for arthralgia, myalgia and sometimes for fatigue.^7^ HCQ treatment reduced systemic interferon‐stimulated gene expression, and improved ESR, IgG, and IgM levels in SS.^8^ HCQ had been reported that can enhance salivary secretion in patients with pSS,^3^, ^6^, ^9^, ^10^, ^11^ however, a systematic review has shown no clear effect of HCQ in improving dry mouth and dry eye in people with pSS.^3^ Therefore, finding a drug that is useful for relieving dryness symptoms and modulating the systemic immune response is of great clinical significance.

Total glucosides of peony (TGP) is obtained from the dried roots of the *Paeonia lactiflora* pall, which has a history more than 1000 years of use as a herbal medicine in China for the treatment of pain, inflammation, and immune disorders.^12^ Some studies have found that the TGP has a wide range of anti‐inflammatory and immunomodulatory functions for rheumatic immune‐related diseases.^12^, ^13^, ^14^ Additionally, a 24‐week multicenter, randomized, double‐blinded, placebo‐controlled clinical trial has showed that TGP can improves dryness of the eyes/throat/vagina, fatigue, mental discomfort, Schirmer*'*s test, erythrocyte sedimentation rate, and salivary flow‐rate values.^6^ An animal study showed that TGP has almost the same efficacy to HCQ in staving off the paroxysm of Sjögren's syndrome‐like disease in nonobese diabetic mice.^15^

It is plausible to combine TGP and HCQ in pSS, and some clinical trials seemed effective, but all of these are all small samples. Meta‐analyses can systematically evaluate and summarize existing relevant studies, leading to more reliable results. Up to date, only two systematic reviews regarding the efficacy and safety of TGP in pSS has been reported.^16^, ^17^ However, the studies included in these two reviews used two different immunosuppressants, HCQ, and methotrexate, as controls. Of these studies, only three or four used HCQ and the most recent one was published in 2013. So, we resystematically reviewed the latest randomized controlled trials (RCTs) to comprehensive assessment for the efficacy and safety of TGP plus HCQ compared to HCQ treatment, thereby aiding clinical decision‐making in the therapy of pSS.

## 2 METHODS

### 2.1 Search strategy

We searched five databases including PubMed, EMBASE, Web of Science, Wan Fang medical database, and the China National Knowledge Internet (CNKI) to obtain all relevant RCTs up to May 10, 2022. We retrieved literatures by controlled vocabularies, like Medical Subject Headings (MeSH), and free‐text vocabularies, including “total glucosides of paeony,” “Baishao Zonggan,” “TGP,” “HCQ,” “Sjögrens Syndrome,” “pSS,” and “pSS.” When searching Chinese databases, we translate these words into Chinese for the same search.

### 2.2 Inclusion criteria and exclusion criteria

#### 2.2.1 Studies were included if they met the following criteria

(1) Study type: RCTs, no matter what the blind method, country, and language.
(2) Patient: all races and genders, aged ≥18 years and diagnosed with pSS. The diagnosis of pSS was consistent with the International Classification of pSS in 2002.^18^
(3) Comparison: use of HCQ, with or without other medicines.
(4) Intervention: treatment was combined with total glycosides of paeony on the basis of the control group.

#### 2.2.2 Studies were excluded if they met the following criteria

(1) The trials did not provide the outcome measures required.
(2) The trials lacking detailed outcome data.
(3) Repeated published trials.

### 2.3 Outcome measures

The primary outcome measures included disappeared clinical symptoms (dry mouth and dry eyes). The secondary outcomes were as follows: Schirmer*'*s test, Saliva flow test, Inflammatory indicators, for example, erythrocyte sedimentation rate (ESR), index of immunoglobulin G (IgG), immunoglobulin M (IgM), immunoglobulin A (IgA), and adverse events (AEs).

### 2.4 Data extraction and study quality assessment

Two review authors (Shilei Chen and Aiping Zhang) independently searched and screened all titles and abstracts according to the criterias set in advance. For articles with potential inclusion, obtain the full text to determine whether to include according to the entry criteria. Data were collected from all included trials and populated into a standardized table drawn specifically for this meta‐analysis. The table contains authors, publication year, basic information about participants, intervention method, duration of treatment, and outcome measurements. When disagreements arose, two researchers interacted with each other or consulted with other authors to make judgments based on consensus. Two independent investigators assessed study quality based on the Cochrane risk of bias assessment tool, it comprises the following items: random sequence generation, allocation concealment, blinding of participants and personnel, blinding of outcome assessment, incomplete outcome data, selective reporting, other bias. “Low risk” and “High risk” were used to determine the standards mentioned above. If it could not be judged whether it is high or low risk, it was determined to be “unclear.”

### 2.5 Statistical analysis

RevMan version 5.4 obtained from the official website was used for statistical analysis. Dichotomous data were calculated using odds risks (OR). Continuous data were calculated using mean differences (MD). All analyses were performed using 95% confidence intervals. Statistical heterogeneity of the studies was inspected by *I* ^2^ test. The heterogeneity is the criterion for selecting a statistical model. The adoption of fixed models needs to satisfy *I* ^2^ < 50%, and when *I* ^2^ over 50%, a random‐effects model was more suitable. *p* < .05 was considered significant for difference and was used for all statistics. When the heterogeneity is beyond reasonable limits, it means that the variation between studies is large and we need to conduct further sensitivity analyses and subgroup studies to determine the source of heterogeneity.

## 3 RESULTS

### 3.1 Characteristics of included studies

**Figure 1:**
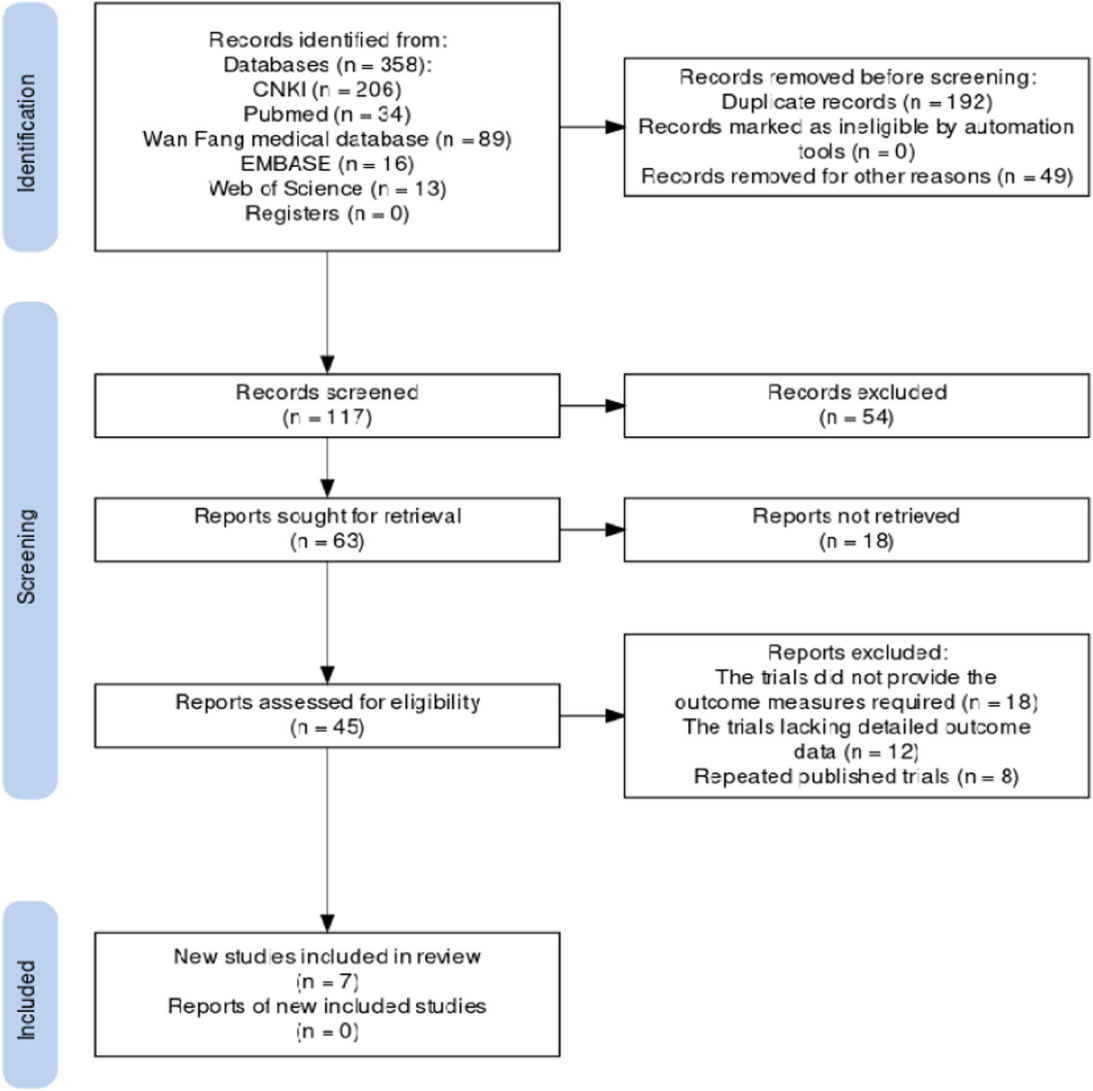
Flow chart of trails selection.

A total of 358 relevant records were retrieved through the preliminary search. After culling out duplicate reported trials, there were 117 trials remained. Based on the above inclusion criteria and exclusion criteria, finally, seven RCTs satisfied the inclusion criteria,^19^, ^20^, ^21^, ^22^, ^23^, ^24^, ^25^ which included 632 patients tested. The included trials ranged in size from 48 to 200 people, with the vast majority being female. HCQ + TGP versus HCQ was used in six of the seven trials. Only one trial used prednisone (PDN) + HCQ + TGP in the intervention arm and PDN + HCQ in the control arm. Flow chart of the screening used in this study is showed in Figure 1. Information about the tests selected for this study are illustrated in Table 1.

**Table 1:** The characteristics of the included trials.

Study	Sample size	Sex (Female)	Age (years)	Intervention measures		Treatment duration (weeks)	Outcomes	Adverse events
	I/C	I/C	I/C	I	C			
He^19^	26/22	26/22	35 ± 12/35 ± 12	HCQ + TGP (0.6 g tid)	HCQ (0.2 g bid)	12	①②④⑤⑥⑦⑧	Occurrence
Lu et al.^20^	50/50	46/47	51.88 ± 10.24/52.4 ± 9.18	PDN + HCQ + TGP (0.6 g tid)	PDN (15 mg qd)+HCQ (0.2 g bid)	12	①②③⑤	Occurrence
Gao^21^	50/50	45/47	53.28 ± 5.06/54.35 ± 5.74	HCQ + TGP (0.6 g tid)	HCQ (0.2 g bid)	12	①④⑤⑥⑦⑧	Not reported
Tang et al.^22^	33/33	—	56.36 ± 8.99/56.42 ± 10.41	HCQ + TGP (0.6 g tid)	HCQ (0.1 g bid)	12	①②⑤	Not reported
Wang^23^	30/30	24/25	50.22 ± 14.76/52.07 ± 15.85	HCQ + TGP (0.6 g tid)	HCQ (0.2 g bid)	12	①⑦	Occurrence
Zhao^24^	30/28	29/26	42.9 ± 11.6/44.5 ± 11.6	HCQ + TGP (0.6 g tid)	HCQ (0.1 g bid)	24	②④⑤⑥⑦⑧	Occurrence
Zhao^25^	100/100	91/91	49. 8 ± 5. 4/50. 1 ± 5. 6	HCQ + TGP (0.6 g tid)	HCQ (0.2 g bid)	48	①⑦	Occurrence

*Note*: I, intervention group; C, control group. ①, Clinical symptoms disappear (dry mouth and dry eyes); ②, erythrocyte sedimentation rate; ③, CRP; ④, immunoglobulin A; ⑤, immunoglobulin G; ⑥, immunoglobulin M; ⑦, Saliva flow test; ⑧ Schirmer's test.

### 3.2 Quality of included studies

**Figure 2:**
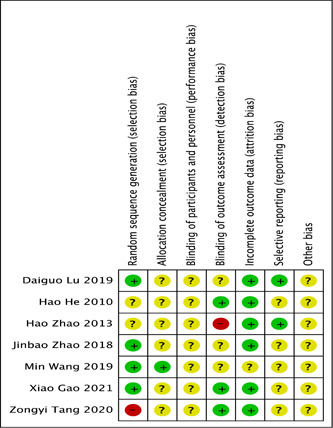
Risk of bias summary of included studies: “?” unclear risk, “+” low risk, and “‐” high risk.

Four of the seven trials reported the method of randomization and were judged as “low risk”; two of the other trials, although reported randomization, they lacked detailed description and were judged as “unclear risk”; the remaining one study was considered as “high risk” because it divided groups by medication. There was only one study reported allocation concealment and except for this experiment, all other experiments were judged to be of unclear risk. No study described the blinding of participants. Three studies described the blinding of outcome assessment, one study was judged as “high risk.” Six studies offered the intact outcome data. All studies were considered to be “low risk” in terms of selective reporting and “unclear risk” of other bias (Figure 2).

### 3.3 Clinical symptoms disappear (dry mouth and dry eyes)

**Figure 3:**
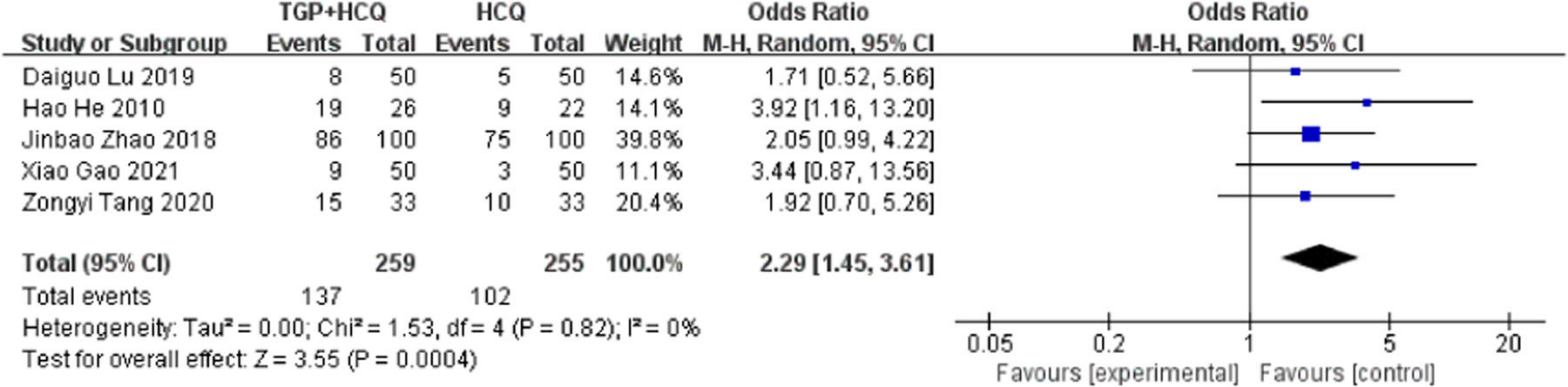
Forest plot of studies comparing total glucosides of paeony (TGP) + hydroxychloroquine (HCQ) group and the HCQ group, examining the effect on relieving clinical symptoms (dry mouth and dry eyes).

There were five studies^19^, ^20^, ^21^, ^22^, ^25^ reporting clinical symptoms disappear (dry mouth and dry eyes). The results of the study showed that the combination of TGP and HCQ can effectively improve clinical symptoms in patients with Sjögren's syndrome (MD = 2.29, 95% confidence interval [CI]: 1.45–3.61, *I* ^2^ = 0%, *p* = .0004), showing combined intervention of TGP and HCQ being more advantageous than single usage of HCQ. The result is as shown below (Figure 3).

### 3.4 ESR

**Figure 4:**
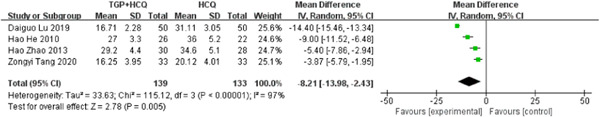
Forest plot of studies comparing total glucosides of paeony (TGP) + hydroxychloroquine (HCQ) group and the HCQ group, examining the effect on erythrocyte sedimentation rate (ESR).

Four studies^19^, ^20^, ^22^, ^24^ reported ESR, there is statistical heterogeneity in the study results (MD = −8.21, 95% CI: −13.98 to −2.43, *I* ^2^ = 97%, *p* = .005). So as to lower the heterogeneity, we have to carry out a subgroup analysis according to the drug dosage of HCQ, which can be divided into two subgroups. The study results of 0.1 g HCQ showed that there was no statistical heterogeneity (MD = −4.45, 95% CI: −5.96 to −2.94, *I* ^2^ = 0%, *p* < .00001); The study results of 0.2 g HCQ showed that there was still heterogeneity, despite a 4% decrease compared to overall heterogeneity. Then, we conducted subgroup analysis depending on duration, which also have two groups. The subgroup results of the 12‐week course showed high heterogeneity, whereas the 24‐week course subgroup includes only one study with a small sample size, and no valid analytical results were available. So, the duration of treatment is not the origin of the high statistical heterogeneity. We further conducted sensitivity analyses and found out its heterogeneity may be mainly due to one study, which explained removing the study about Daiguo Lu 2019, the heterogeneity reduced to 80%. Both pooled and subgroup analyses showed that the combination of TGP and HCQ reduced ESR in patients more than HCQ alone. The result is as shown below (Figure 4; Supporting Information: Figures S1–S3).

### 3.5 IgM

**Figure 5:**
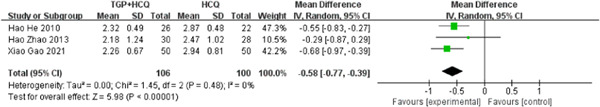
Forest plot of studies comparing total glucosides of paeony (TGP) + hydroxychloroquine (HCQ) group and the HCQ group, examining the effect on immunoglobulin M.

There were three studies^19^, ^20^, ^21^, ^24^ reporting IgM. The results of the study show that the addition of TGP to HCQ can significantly reduce the serum IgM level of patients (MD = −0.58, 95% CI: −0.77 to −0.39, *I* ^2^ = 0%, *p* < .00001), suggesting that when TGP is used in combination with HCQ, there are differences in IgM effect accordingly. The result was shown below (Figure 5).

### 3.6 IgG

**Figure 6:**
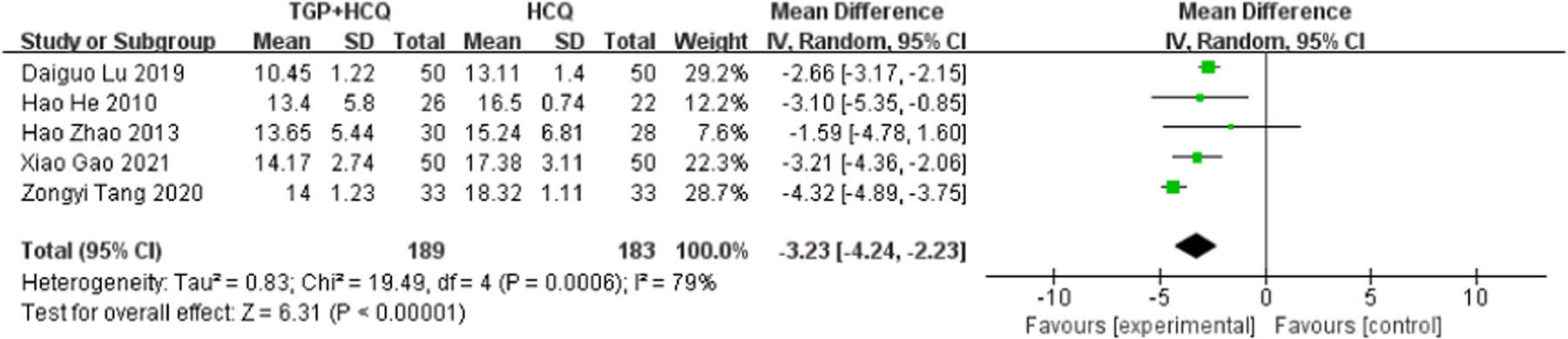
Forest plot of studies comparing total glucosides of paeony (TGP) + hydroxychloroquine (HCQ) group and the HCQ group, examining the effect on immunoglobulin G (IgG).

There were five studies^19^, ^20^, ^21^, ^22^, ^24^ reporting IgG. The test for heterogeneity showed large heterogeneity between the literature (MD = −3.23, 95% CI: −4.24 to −2.23, *I* ^2^ = 79%, *p* < .00001). To minish the heterogeneity, we have to implement subgroup analysis. We assign five studies to two groups based on the drug dosage of HCQ. The study results of 0.1 g HCQ showed that there was still statistical heterogeneity, despite a 16% decrease compared to overall heterogeneity. The study results of 0.2 g HCQ showed that there is no statistical heterogeneity (MD = −2.27, 95% CI: −3.23 to −2.31, *I* ^2^ = 0%, *p* < .00001). Then, we carried out subgroup analysis based on the course of treatment, which can be also divided into two subgroups. The subgroup results of the 12‐week course still showed large statistical heterogeneity (MD = −3.37, 95% CI: −4.42 to −2.32, *I* ^2^ = 84%, *p* < .00001), whereas the 24‐week course subgroup had only one group of Hao Zhao 2013, and no valid analytical results were available. Therefore, the difference in the course of treatment is not the cause of high heterogeneity. We further conducted sensitivity analyses and found out its heterogeneity may be mainly due to one study, which explained removing the study about Zongyi Tang 2020, the heterogeneity disappeared (*I* ^2^ = 0). Both pooled and subgroup analyses showed that the combination of TGP and HCQ reduced IgG in patients more than HCQ alone. The result was shown below (Figure 6; Supporting Information: Figures S4–S6).

### 3.7 IgA

**Figure 7:**
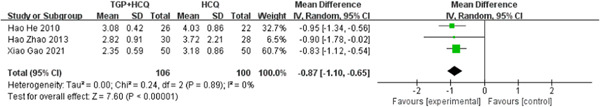
Forest plot of studies comparing total glucosides of paeony (TGP) + hydroxychloroquine (HCQ) group and the HCQ group, examining the effect on immunoglobulin A (IgA).

There were three studies^19^, ^21^, ^23^ reporting IgA. The results of the study show that TGP + HCQ can significantly reduce the serum IgA level of patients (MD = −0.87, 95% CI: −1.10 to −0.65, *I* ^2^ = 0%, *p* < .00001). The result was shown below (Figure 7).

### 3.8 Schirmer*'*s test

**Figure 8:**
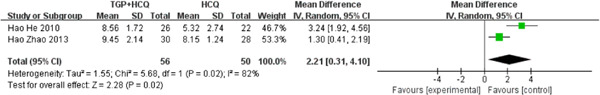
Forest plot of studies comparing total glucosides of paeony (TGP) + hydroxychloroquine (HCQ) group and the HCQ group, examining the effect on Schirmer's test.

Two studies^19^, ^24^ reported the Schirmer*'*s test. The results of the study show that TGP + HCQ can increase tear production levels of patients (MD = 2.21, 95% CI: 0.31–4.10, *I* ^2^ = 82%, *p* = .02). Due to the small number of studies, we were unable to perform the next step of sensitivity analysis as well as subgroup analysis to look for sources of heterogeneity. The result is as shown below (Figure 8).

### 3.9 Salivary flow rate

**Figure 9:**
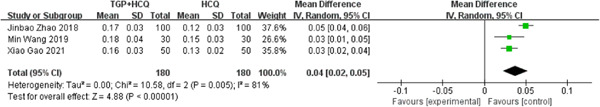
Forest plot of studies comparing total glucosides of paeony (TGP) + hydroxychloroquine (HCQ) group and the HCQ group, examining the effect on static salivary flow rate.

**Figure 10:**
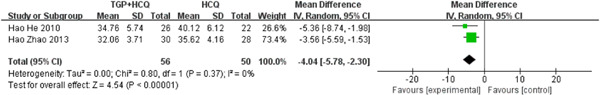
Forest plot of studies comparing total glucosides of paeony (TGP) + hydroxychloroquine (HCQ) group and the HCQ group, examining the effect on dynamic saliva flow rate.

There are five studies^19^, ^21^, ^23^, ^24^, ^25^ reporting salivary flow rate. Three of the studies measured static salivary flow rate (salivary flow rates calculated by measuring the amount of saliva in 15 min), while the other two studies measured dynamic salivary flow rate (measuring the time taken to melt the sugar cubes), so we analyzed them separately. The results showed that TGP combined with HCQ increased the static salivary flow rate compared to HCQ alone (MD = 0.04, 95% CI: 0.02–0.05, *I* ^2^ = 81%, *p* < .00001). Due to the high heterogeneity, we performed a sensitivity analysis and found that the heterogeneity disappeared after excluding the study by Jinbao Zhao 2018. This may be due to the large sample size of this study. Also, the results showed that TGP combined with HCQ reduced the time required to melt the sugar cubes (MD = −4.04, 95% CI: −5.78 to −2.30, *I* ^2^ = 0%, *p* < .00001), that is increased the dynamic saliva flow rate, compared to HCQ alone. The result is as shown below (Figures 9 and 10; Supporting Information: Figure S7).

### 3.10 AEs

**Figure 11:**
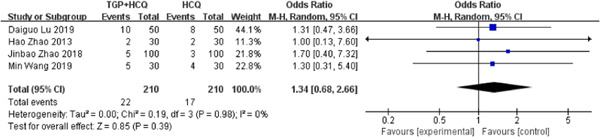
Forest plot of studies comparing total glucosides of paeony (TGP) + hydroxychloroquine (HCQ) group and the HCQ group, examining the effect on AEs.

There are four studies^20^, ^23^, ^24^, ^25^ reporting adverse events. According to the results of the study, compared with HCQ alone, TGP + HCQ caused more adverse events in patients with pSS, like diarrhea, vomit, and bloating (MD = 1.34, 95% CI: 0.68–2.66, *I* ^2^ = 0%, *p* = .39). The result is as shown below (Figure 11).

## 4 DISCUSSION

All published studies reporting the efficacy and safety of combination of TGP and HCQ in treating pSS draw their data from China. The majority reported combination of TGP and HCQ, while Lu et al. reported combination of PDN, HCQ, and TGP. A combination of TGP and HCQ had often been found to be more effective in reducing clinical symptoms, serum IgM, IgG, and IgA, although with varying magnitudes.

This systematic review and Meta‐analysis of such studies corroborated the efficacy of combination of TGP and HCQ to be significantly elevated when compared with single HCQ. Most cases enrolled were women, predominantly perimenopausal or postmenopausal, which may be due to higher pSS incidence at a ratio of 9:1 compared to men.^26^ More studies are required to demonstrate the extent of any effectiveness differences between genders or ages. Many hypotheses have been proposed to explain the overall marked sex bias in pSS.^27^

HCQ has been reported no superior to placebo in alleviating dry mouth, dry eyes, or Schirmer test.^3^ TGP may have a unique advantage in improving lacrimal gland secretion function, and can exert this effect advantage during its combined use. A Meta‐analysis showed that significant difference in Schirmers test comparing TGP with placebo (PBO) (*p* < .00001), or TGP + HCQ with HCQ (*p* = .0006).^16^ Our Meta‐analysis demonstrated that TGP + HCQ was superior to HCQ alone in reducing clinical symptoms(dry mouth or dry eyes), which was consistent with previous study.^16^ In addition, clinical examination results were consistent with the patient's clinical symptoms. Analyses showed that TGP + HCQ was more effective than HCQ alone in improving the patient's Schirmer test and salivary flow rate test. It can be seen that the TGP is important in promoting glandular secretion.

According to the meta‐analysis published by Wang et al.,^3^ HCQ could reduce ESR compared with PBO. Our pooled data indicated that TGP + HCQ is superior to HCQ alone in reducing ESR. Subgroup analysis suggested that Lu et al.^20^ is a source of the statistical heterogeneity, which showed PDN + HCQ + TGP is superior to PDN + HCQ in reducing ESR. Meanwhile, by further subgroup analyses, we found that ESR did not decrease further when the dosing regimen was prolonged, while ESR decreased more significantly when the dose was increased. However, there are fewer relevant studies, and it is not possible to clarify the correlation between the dose of TGP and the magnitude of ESR decline for the time being. To be sure, TGP + HCQ can improve inflammatory indices in pSS. This may be related to the fact that TGP can inhibit the generation of pro‐inflammatory mediators,^28^ promote the production of interleukin‐4 and interleukin‐10 and reduce the production of interleukin‐2 and interleukin‐17 in the serum and lymphocyte supernatant,^12^ which leads to anti‐inflammatory effect.

Previous studies have shown that HCQ can significantly reduce immunoglobulins in pSS patients.^8^, ^10^ A meta‐analysis displayed significant differences in serum γ‐globulin, IgG, IgA, and IgM (*p* ≤ .01) in the coadministration of TGP with IS compared with IS alone.^28^ Our meta‐analysis indicated that TGP + HCQ is superior to HCQ alone in improving three kinds of immunoglobulins (IgG, IgA, and IgM). This suggested that TGP may have the synergistic effect on enhancing the immunomodulatory activity of HCQ.

Our review also displayed a more detailed safety assessment of TGP + HCQ than the previous analysis. Comparing TGP + HCQ with HCQ alone, there were insignificant differences in AEs (*p* > .05). In addition to the common AEs like transaminase abnormality and blurred vision when HCQ used alone, diarrhea is still the manifestation. However, AEs in some of the patients were gradually relieved on the completion of the experiment without treatment in these included trials. This means that we can combine TGP and HCQ to treat pSS in clinic without increasing safety risk. The dose of TGP used in the seven trials was 1800 mg/day, while HCQ varied from 0.1 to 0.2 g/day. More in‐depth analyses are needed if there are more high quality trials in future, to determine whether effectiveness and safety of TGP + HCQ are in dose depending. In view of this, more multi‐center and large‐scale RCTs are in greater demand for a more complete and objective evaluation of the efficacy and safety of in patients with pSS in the foreseeable future.

It is now generally accepted that the pathogenesis of pSS is a multistep process, triggered by environmental factors, with the intrinsic immune system triggering the initial event, and a continuing interaction between the intrinsic and adaptive immune systems leading to an autoimmune outbreak. The result is the stimulation of auto‐reactive B cells, the production of autoantibodies, and chronic inflammation of the salivary and lacrimal glands and other tissues.^29^ HCQ interferes with lysosomal enzymatic actions, major histocompatibility complex class‐II (MHC‐II)‐mediated antigen presentation, and toll‐like receptor (TLR) functions.^30^ TGP is a novel bidirectional immunomodulator possessing multiple pharmacological activities including antioxidant, antiapoptotic, and anti‐inflammatory. Pae Paeoniflorin (Pae) is the major active component of TGP. Pae could balance the subsets of immune cells through inhibiting abnormal activated cell subsets and restoring regulatory cell subsets. Pae could reduce the expression of inflammatory factors and ameliorating inflammatory damage in exocrine gland tissues leads to the treatment of pSS by regulating signaling pathways (GPCR pathway, MAPKs/NF‐κB pathway, PI3K/Akt/mTOR pathway, JAK2/STAT3 pathway, TGFβ/Smads, and etc.).^12^ Synergism of HCQ and TGP, instead of antagonism, indicates that they bind with different receptors and regulate different signaling pathways.

The current treatment of pSS is inseparable from immunosuppressants. Immunosuppressants such as HCQ tend to have a range of side effects, including increased risk of infection and impaired liver and kidney function. Our research suggests that TGP and HCQ may act on different biological pathways, and the combination of the two has a synergistic effect. TGP has the advantages of immune regulation, mild effect, many indications, and few adverse drug reactions. If TGP can effectively enhance the efficacy of immunosuppressants, it may help reduce drug dosage and these side effects. And in China, TGP has been widely used to treat autoimmune diseases, including inflammatory arthritis, systemic autoimmune diseases, and skin diseases. In the future, TGP may be used for patients with pSS in more countries and regions.

### 4.1 Limitations

In this review, we have some limitations to be aware of. First, only seven trials met the inclusion criteria, and all these trials were implemented in China. This limitation causes out inability to precisely evaluate the role of TGP + HCQ in patients with pSS. Second, the quality assessment of the methodology exhibited defects: one trial (14.3%) ran a high risk of bias in blinding of participants because it divided groups by medication, one trials (14.3%) ran a high risk of bias in blinding of outcome assessment. Third, the random effect model was used in several outcome analyses due to high heterogeneity, which may be related to different doses and intervention duration of TGP + HCQ. Fourth, although eight outcome measurements were chosen in “TGP + HCQ” versus HCQ alone, some indexes, such as ESSDAI and ESSPRI were not mentioned, and some like Schirmers test were only reported in two articles. Fifth, the included trials of 3–6 months can not provide long‐term outcome reports, we have not known whether to prolong the time would observe AEs more clearly. These inadequacies should be taken into account when analyzing the results. Thus, there is a pressing demand for high‐quality, large‐scale RCTs to evaluate the efficacy and safety of TGP + HCQ in patients with pSS.

## 5 CONCLUSION

As a widely used statistical method, meta‐analysis can integrate all relevant studies, explore the consistency and differences between individual studies, and obtain statistical analysis results that are closer to the real situation. The aim of this review is to assess the clinical efficacy and safety of TGP combined with HCQ in the treatment of pSS through the meta‐analysis. Evidence from this meta‐analysis suggests that HCQ and TGP are synergistic in the treatment of pSS. The addition of TGP compared to HCQ alone can not only more effectively improve the clinical dryness symptoms of patients, promote the secretion of the tear and salivary glands, but also reduce the blood inflammation index and immunoglobulin index. And it is worth noting that the addition of TGP did not significantly increase the incidence of severe adverse events. Therefore, TGP combined with HCQ is effective and safe for the treatment of pSS.

## AUTHOR CONTRIBUTIONS

**Aiping Zhang**: Data curation; formal analysis; investigation; writing—original draft. **Shilei Chen**: Data curation; methodology; project administration; writing—original draft. **Riyang Lin**: Writing—review and editing.

## CONFLICT OF INTEREST STATEMENT

The authors declare no conflict of interest.

## Supporting information

Supp Figure 1: Sensitivity analysis: heterogeneity decreased to 80% after removing the study of Daiguo Lu 2019.

Supp Figure 2: Forest plot for subgroup analysis based on hydroxychloroquine dose grouping, examining the effect on erythrocyte sedimentation rate.

Supp Figure 3: Forest plot for subgroup analysis grouped according to treatment duration, examining the effect on erythrocyte sedimentation rate.

Supp Figure 4: Sensitivity analysis: heterogeneity disappeared after removing the study of Zongyi Tang 2020.

Supp Figure 5: Forest plot for subgroup analysis based on hydroxychloroquine dose grouping, examining the effect on immunoglobulin G.

Supp Figure 6: Forest plot for subgroup analysis grouped according to treatment duration, examining the effect on immunoglobulin G.

Supp Figure 7: Sensitivity analysis: heterogeneity disappeared after removing the study of Jinbao Zhao 2018.

## Data Availability

Data could be obtained from the corresponding author.
