# Supplementary figures and images for: Combined use of total glucosides of paeony and hydroxychloroquine in primary Sjögren's syndrome: A systematic review
Source: Immun Inflamm Dis. 2023 Oct 20;11(10):e1044. doi: 10.1002/iid3.1044 (PMC10587734; doi:10.1002/iid3.1044)

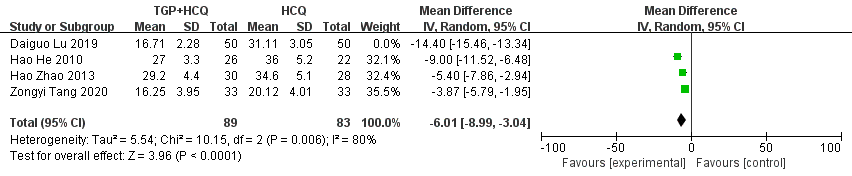

Supplement: Supplementary file 1 — Supp Figure 1: Sensitivity analysis: heterogeneity decreased to 80% after removing the study of Daiguo Lu 2019. [file IID3-11-e1044-s003.png]

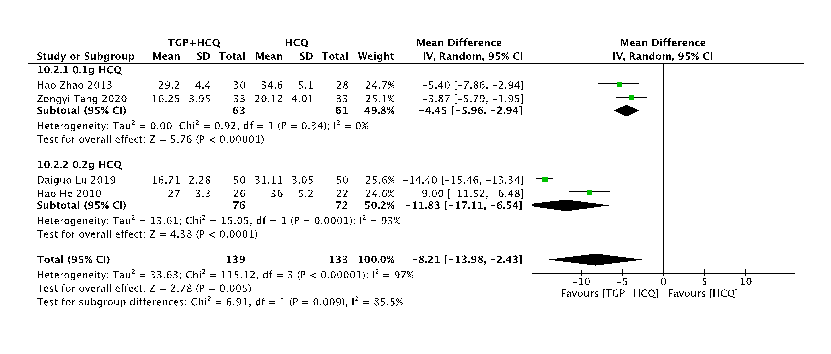

Supplement: Supplementary file 2 — Supp Figure 2: Forest plot for subgroup analysis based on hydroxychloroquine dose grouping, examining the effect on erythrocyte sedimentation rate. [file IID3-11-e1044-s006.png]

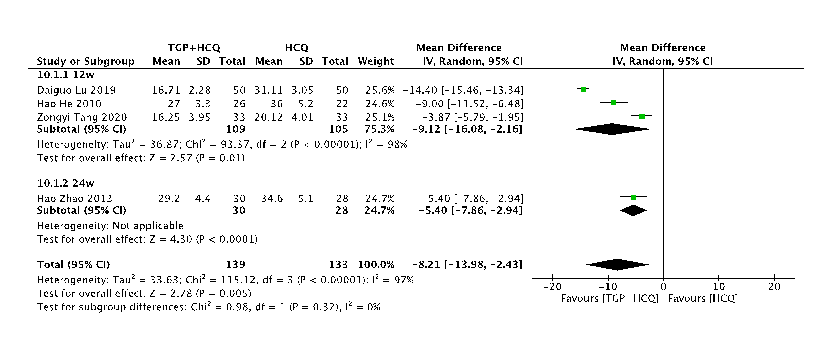

Supplement: Supplementary file 3 — Supp Figure 3: Forest plot for subgroup analysis grouped according to treatment duration, examining the effect on erythrocyte sedimentation rate. [file IID3-11-e1044-s007.png]

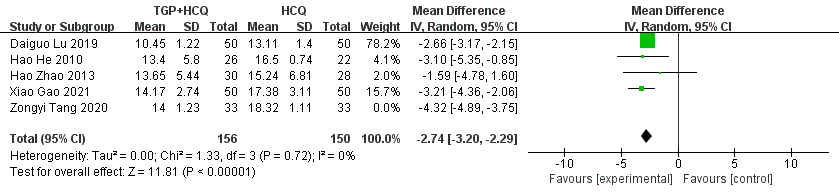

Supplement: Supplementary file 4 — Supp Figure 4: Sensitivity analysis: heterogeneity disappeared after removing the study of Zongyi Tang 2020. [file IID3-11-e1044-s004.png]

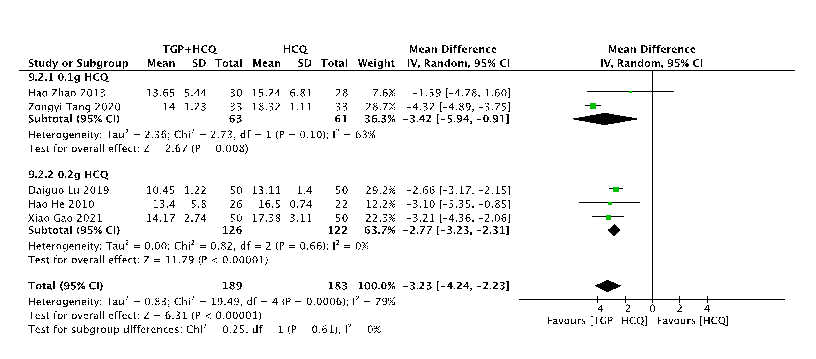

Supplement: Supplementary file 5 — Supp Figure 5: Forest plot for subgroup analysis based on hydroxychloroquine dose grouping, examining the effect on immunoglobulin G. [file IID3-11-e1044-s002.png]

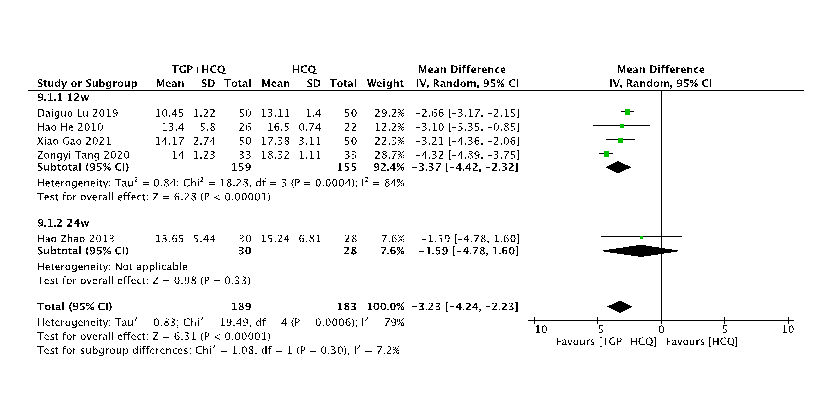

Supplement: Supplementary file 6 — Supp Figure 6: Forest plot for subgroup analysis grouped according to treatment duration, examining the effect on immunoglobulin G. [file IID3-11-e1044-s001.png]

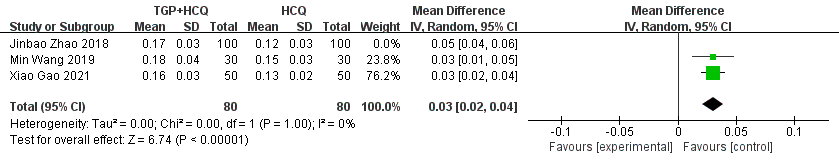

Supplement: Supplementary file 7 — Supp Figure 7: Sensitivity analysis: heterogeneity disappeared after removing the study of Jinbao Zhao 2018. [file IID3-11-e1044-s005.png]
